# SprayNPray: user-friendly taxonomic profiling of genome and metagenome contigs

**DOI:** 10.1186/s12864-022-08382-2

**Published:** 2022-03-12

**Authors:** Arkadiy I. Garber, Catherine R. Armbruster, Stella E. Lee, Vaughn S. Cooper, Jennifer M. Bomberger, Sean M. McAllister

**Affiliations:** 1 Biodesign Center for Mechanisms of Evolution, Arizona State University, Tempe, AZ 85287 USA; 2 Department of Microbiology and Molecular Genetics, School of Medicine, University of Pittsburgh, Pittsburgh, PA 15219 USA; 3 Department of Otolaryngology, University of Pittsburgh Medical Center, Pittsburgh, PA 15213 USA; 4 Pacific Marine Environmental Laboratory, National Oceanic and Atmospheric Administration, Seattle, WA 98115 USA; 5 The Cooperative Institute for Climate, Ocean, and Ecosystem Studies, University of Washington, Seattle, WA 98105 USA

**Keywords:** Symbiont, Taxonomic classification, Binning, Bioinformatics, Contaminant identification, HGT, Horizontal gene transfer

## Abstract

**Background:**

Shotgun sequencing of cultured microbial isolates/individual eukaryotes (whole-genome sequencing) and microbial communities (metagenomics) has become commonplace in biology. Very often, sequenced samples encompass organisms spanning multiple domains of life, necessitating increasingly elaborate software for accurate taxonomic classification of assembled sequences.

**Results:**

While many software tools for taxonomic classification exist, SprayNPray offers a quick and user-friendly, semi-automated approach, allowing users to separate contigs by taxonomy (and other metrics) of interest. Easy installation, usage, and intuitive output, which is amenable to visual inspection and/or further computational parsing, will reduce barriers for biologists beginning to analyze genomes and metagenomes. This approach can be used for broad-level overviews, preliminary analyses, or as a supplement to other taxonomic classification or binning software. SprayNPray profiles contigs using multiple metrics, including closest homologs from a user-specified reference database, gene density, read coverage, GC content, tetranucleotide frequency, and codon-usage bias.

**Conclusions:**

The output from this software is designed to allow users to spot-check metagenome-assembled genomes, identify, and remove contigs from putative contaminants in isolate assemblies, identify bacteria in eukaryotic assemblies (and vice-versa), and identify possible horizontal gene transfer events.

**Supplementary Information:**

The online version contains supplementary material available at 10.1186/s12864-022-08382-2.

## Background

There is particular demand among biologists for taxonomic classification and partitioning of genome and metagenome assemblies. In particular, there is a need for easy-to-use tools that allow novice users to more efficiently begin these analyses without sophisticated knowledge of programming languages like Python. Tools exist for taxonomic classification and tree-building, including those that use sets of specific gene markers on genomes or metagenome-assembled genomes (MAGs) binned from an original assembly (Table 1). The partitioning of contigs (i.e. binning) is often carried out independently of taxonomic-classification, and takes into account sequence compositional data (GC-content and tetranucleotide frequency) and read coverage; however, open-source software is available that can incorporate taxonomic/phylogenetic information to aid binning (Table 1). While many tools exist for classification of sequenced samples, several barriers exist for novice users, including the choice of which tools to use, how to use them, and the ability to efficiently iterate through these tools when analyzing multiple samples.

**Table 1:** Summary of published software for taxonomic classification and binning

*Overall purpose*	*Software*	*Citation*
*Taxonomic classification and tree-building (using specific markers)*	CheckM	[1]
	PhyloSift	[2]
	GToTree	[3]
	phyloSkeleton	[4]
*Taxonomic classification at the contig level*	Kaiju	[5]
	Kraken	[6]
	CLARK	[7]
	FOCUS	[8]
	MEGAN	[9]
	BlobTools	[10]
*Taxonomic classification at the contig level (using all predicted genes)*	CAT	[11]
	Mmseqs2	[12]
*Binning with sequence compositional data*	MetaBAT	[13]
	MaxBin	[14]
	Concoct	[15]
	BinSanity	[16]
	VizBin	[17]
*Binning with sequence compositional data and taxonomic information*	Anvi’o	[18]
	BlobTools	[10]

Here, we present an open-source bioinformatics tool, SprayNPray, that combines taxonomic/phylogenetic information with a variety of other metrics for each contig (discussed in detail below), allowing users to manually or automatically group contigs based on these metrics. This software wraps together several steps, which would otherwise require more advanced knowledge of a coding language to efficiently iterate through, and processes the output in a way that allows for easy visual inspection, manual curation, and/or further computational parsing. It is also organized in a way to allow for identification of pathogens, symbionts, and horizontally acquired genes in eukaryotic assemblies. The case studies presented below demonstrate the software’s versatility and potential usefulness to biologists dealing with non-axenic samples.

## Implementation

**Fig. 1:**
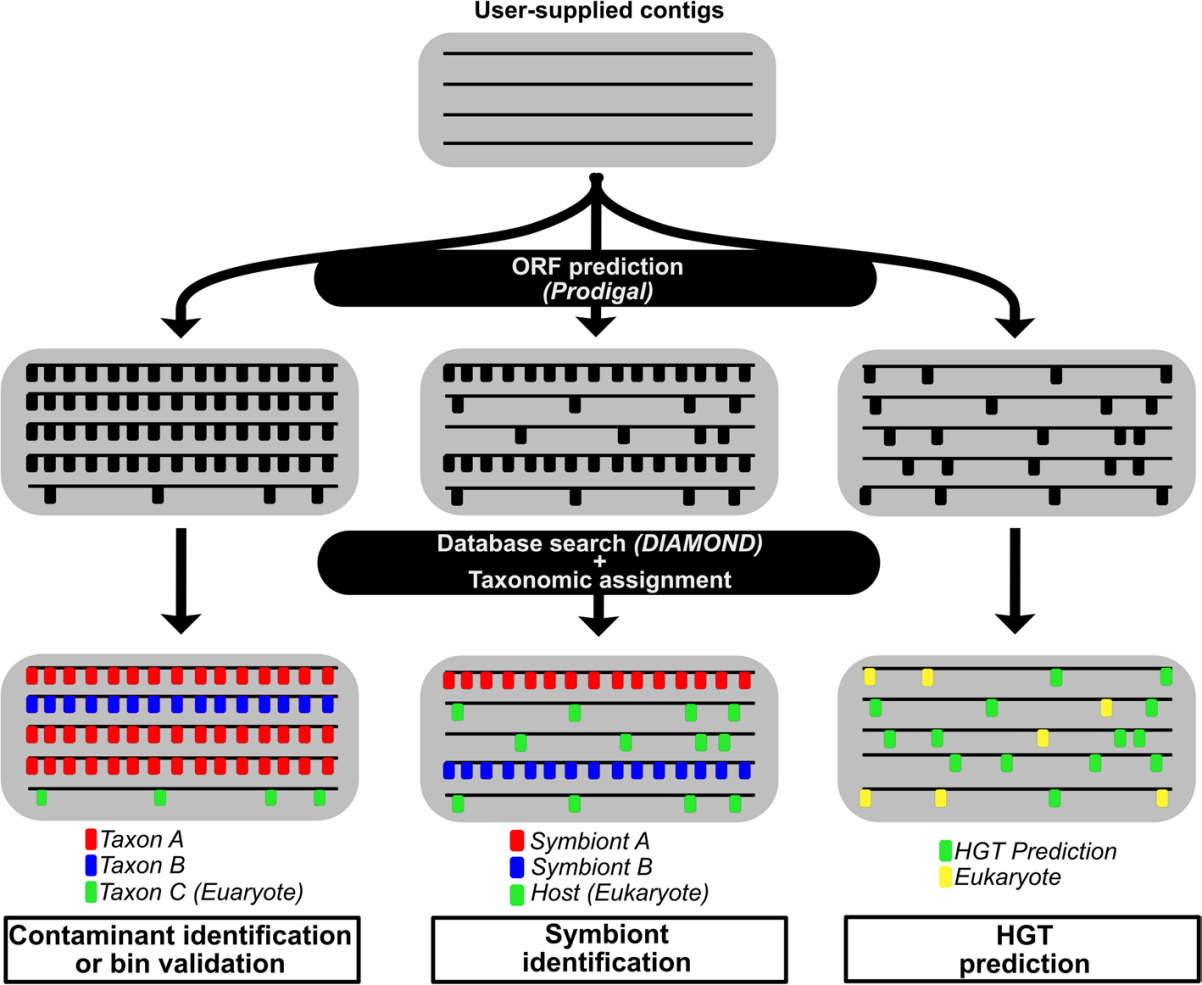
Overall workflow of the SprayNPray pipeline, with the four different uses (contaminant identification, bin validation, symbiont identification, and HGT prediction) shown. Horizontal lines in each gray box represent contigs, while the smaller vertical lines perpendicular to the contigs represent ORFs

SprayNPray, implemented in Python (version 3), is an easy-to-use software that provides a broad overview of input contigs by comparing each predicted ORF against a user-set reference database (recommended: NCBI’s non-redundant [nr] protein database [ftp://ftp.ncbi.nih.gov/blast/db/FASTA]). SprayNPray requires two inputs, a FASTA file of contigs (files with extension “.fna”, according to NCBI’s naming standards for genome FASTA sequences, but also ".fa" and ".fasta" in some cases) and a user-defined reference database, preferably NCBI’s non-redundant (nr) database of proteins. Users also have the option of providing a BAM file containing read coverage information; in this case, a script from the MetaBAT package (jgi_summarize_bam_contig_depths) is used to calculate the average read depth per contig [13]. After predicting ORFs with Prodigal [19], SprayNPray runs DIAMOND v2.0.4.142 [20] to query each ORF against the reference database (Fig. 1). Results of this search are then grouped and written to a spreadsheet, where each row corresponds to a separate contig, followed by the taxonomic affiliation of the top DIAMOND hit to each ORF on that contig. SprayNPray ultimately writes three (optionally, four) output files:

1. In the main output file, SprayNPray provides the following metrics related to the user-supplied contigs:
  **Average amino acid identity (AAI) between the contig ORFs and closest matches in reference:** this will provide users with an idea of how closely related their sequenced organisms are to what currently exists in public databases.
  **Number of genes normalized to the contig length:** bacterial and archaeal genomes are typically gene-dense (~ 1 gene per kbp), compared to eukaryotic genomes (0.9 genes per kbp in some fungi to ~ 0.01 genes per kbp in plants and animals). Further, Prodigal is designed for prokaryotic ORF prediction, leading to suboptimal eukaryotic ORF prediction and subsequent lower gene density estimates. Thus, users can use coding density to deduce bacterial from eukaryotic contigs (Fig. 1).
  **GC-content:** if the provided contigs have organisms of varying levels of GC content, this will allow users to separate sequences based on that metric.
  **Read coverage:** (this metric outputs only if a BAM file is provided): read coverage is useful for separating sequences if organisms represented among the contigs have varying levels of abundance, resulting in different read coverages estimates.
  **Contig length:** contig length is a useful metric for filtering out low-quality contigs, or those too short for binning.
  **Cluster affiliation:** contigs are clustered into putative bins. Cluster/bin assignments are derived from hierarchical clustering of contigs based on tetranucleotide frequency and codon usage bias. The number of clusters is estimated from the average number of hmmsearch (v.3.1b2, [21]) hits per single-copy gene (using the ‘Universal_Hug_et_al’ set of 16 genes available in the GToTree package [3]).
2. SprayNPray provides an R-generated word cloud that is based on the distribution of the top taxonomic hits to genes predicted from the provided contigs (Supplemental Figure 1). When users provide a BAM file along with their contigs, the word sizes in the word cloud are corrected with read coverage information.
3. SprayNPray provides a file containing the top user-specified number of hits (default = 100) to each ORF, allowing users to assess the taxonomic and functional distribution of top homologs to each gene.
4. SprayNPray has the capacity to write FASTA files that represent subsets of the provided contigs. This capability allows users to easily extract contigs belonging to organism(s) of interest (e.g. contaminants, pathogens, symbionts, certain genera). Subsets are created based on user-specified parameters, including: GC-content, coding density, amino acid identity, contig length, read coverage, and cluster/bin affiliation. Additionally, users can directly specify a taxonomic group of interest, and SprayNPray will write a FASTA file containing only contigs where some user-specified percentage (e.g. > 50%) of DIAMOND hits are to that specified taxa. In the event that the parameters by which FASTA files need to be written are unknown prior to running SprayNPray, users can re-run the program, with newly specified parameters (inferred from visually inspecting the output file from a preceding run), with greatly reduced runtime by providing the DIAMOND BLAST output file (file with extension ".blast") from the previous run.
5. When running SprayNPray on an assembly containing eukaryotic contigs, users can also direct the program to specifically look for potential horizontal gene transfers (HGTs) from Bacteria or Archaea to Eukaryota. In this case, SprayNPray will write a separate output file containing putative HGTs. To identify ORFs of possible bacterial or archaeal origin, SprayNPray evaluates the taxonomic distribution of the top user-specified (default = 100) DIAMOND matches for each ORF on each eukaryotic contig, and if more than a user-specified percentage (default 50%) of the hits are to bacterial proteins, that ORF is flagged as a potential HGT of bacterial or archaeal origin. In order for this part of the software to function properly, users need to be sure to include a reference database that encompasses protein sequences from all domains of life (e.g. nr).

## Results

### Case study: simulated metagenome

To demonstrate SprayNPray’s capacity to efficiently and accurately summarize contigs, and extract contigs relevant to species/genera/domains of interest, we ran the software on a fully controlled dataset consisting of a concatenated set of 15 bacterial isolate genomes, 1 archaeal isolate genome, 2 phage genomes, and the genome of unicellular eukaryote *Sphaeroforma arctica* (Supplemental Table 1). Bacterial isolate genomes included the genomes of two closely related species of *Shewanella*, as well as two closely related species of *Geobacter*. To simulate the contig fragmentation that is commonly observed in metagenomic assemblies, we manually broke up contigs longer than 1 Mb into 100 kb fragments. Contigs from each assembly were then concatenated into a single file. This combined file was then used as input to SprayNPray. For 11 of the bacterial genomes and 1 archaeal genome, we selected for each individual isolate by setting the ‘-genus’ flag, requiring that more than 50% of hits to each contig consist of the selected genus. For the two *Shewanella* and two *Geobacter* species, we additionally set the ‘-species’ flags, with the same > 50% threshold, to specify the exact species of interest. The *Bacillus* and *Caulobacter* phage contigs were extracted by setting the ‘-genus’ flags to either ‘*Bacillus’* and ‘*Caulobacter*’, along with the ‘--phage’ flag. To extract contigs corresponding to *S. arctica*, we set the ‘-domain’ flag to ‘Eukaryota’, maximum GC-content to 50%, and maximum coding density to 0.5 genes per kb; the SprayNPray output file from this run (Supplemental File 1) was inspected prior to extracting the *S. arctica* contigs, to confirm that none had a GC-content higher than 50%, or coding density higher than 0.5 genes per kb.

Results demonstrate that the top taxonomic hits to each contig match closely the genome from each contig is derived (Supplemental File 1); although, it is worth noting that since many of these isolate genomes represent model organisms, they are well-represented in the reference database used (nr). Analysis of unpublished data that represents underrepresented organisms is likely to yield results that are more ambiguous (e.g. see *Case study: bin validation*). Using SprayNPray’s ‘--fa’ flag, we were able to generate 19 FASTA files, each of which contained 100% of the contigs belonging to a single isolate genome that was part of the mock community (Supplemental Files 2, 3, 4, 5, 6, 7, 8, 9, 10, 11, 12, 13, 14, 15, 16, 17, 18, 19 and 20). The exact flags and thresholds that were used to extract each genome is listed in Supplemental Table 1.

We used MetaBAT to generate bins from the simulated assembly using all five of MetaBAT’s specificity/sensitivity settings. Overall, MetaBAT performed well in binning genomes from the simulated metagenome, although certain bins appeared contaminated with contigs from a closely related (e.g. Class-level) genome (Supplemental File 21). Moreover, there was no resolution observed between the closely-related genomes of *Geobacter sulfurreducens* and *G. metallireducens*, as well as between *Shewanella oneidensis* and *S. denitrificans*. In other words, a single bin was generated for the two *Geobacter* species, and another single bin for the two *Shewanella* species.

### Case study: bin validation

SprayNPray was used to profile a subset of metagenome bins from the North Pond aquifer [22] using NCBI’s nr database (release 200) as reference. Metagenome-assembled genomes (MAGs) from the North Pond aquifer were obtained from the FigShare link provided by Tully et al. [22]: (https://figshare.com/s/939160bb2d4156022558). To simulate the SprayNPray run as if it were conducted on an unpublished metagenome, we removed top hits corresponding to proteins generated from the original publication [22]. The results (Supplemental Files 22, 23, 24 and 25) demonstrate SprayNPray’s capacity to 1) provide a rough taxonomic prediction of a genome bin and 2) demonstrate that the apparent taxonomic heterogeneity of a bin is due not to contaminating contigs, but to the low representation of a genome in NCBI’s nr database. This deduction is supported by the low average AAI between the ORFs predicted in each contig and their closest hits in NCBI’s RefSeq database (Supplemental Files 22 and 23). Nonetheless, a subset of genome bins with higher similarities (> 85%) to reference proteins recruited a more taxonomically homogenous set of homologs from the reference database (Supplemental Files 24 and 25), and appear less “contaminated”.

### Case study: contaminant identification in cultured isolates

SprayNPray can be used to identify putative contaminants in isolate assemblies. This software was used to remove contaminating *Serratia marcescens* contigs from a genome assembly of *Pseudomonas aeruginosa*, isolated from clinical specimens following an IRB-approved protocol (STUDY19100149) at the University of Pittsburgh [23]. Specimens were streaked onto *Pseudomonas* Isolation agar (PIA), a *Pseudomonas*-selective media on which *Serratia marcescens* and some other species can also grow, and incubated at 37˚C for 48 h. Single colonies were stored as a 30% glycerol stock at -80˚C. Genomic DNA was extracted using a QIAgen DNeasy kit (Qiagen, Hilden, Germany) and sequenced on an Illumina NextSeq 500. Reads were assembled using SPAdes v3.11.0 [24] and contigs smaller than 1kbp were removed.

Visual inspection of SprayNPray’s output revealed the presence of contigs from multiple species (Supplemental Files 26, 27 and 28). Running SprayNPray on these assemblies with the ‘-genus’ flag set to ‘*Pseudomonas’ *created new FASTA files with only sequences corresponding to the *Pseudomonas aeruginosa* (Supplemental Files 29, 30 and 31). In this run, we set the ‘-perc’ flag to 50%, meaning that if more than 50% of the genes on each contig recruited a *Pseudomonas*-related gene as a top hit, then they would be classified as *Pseudomonas*, and written to a separate FASTA file, while contigs that did not meet this criteria were automatically written to a second FASTA file that contained contaminating contigs, which were affiliated with *Serratia* (Supplemental Files 32, 33 and 34). Subsequent analysis with CheckM v1.1.1 [1] confirmed the lack of contamination in the FASTA files written by SprayNPray (Supplemental File 35), while the completeness score for the newly written, clean *Pseudomonas* assemblies remained > 99%, indicating that none of the *Pseudomonas*-sequences were likely removed. It is worth noting, however, that CheckM uses a set of marker genes for completeness estimation. Thus, this completion metric is based solely on those contigs that encode the marker genes. Nonetheless, the file containing *Pseudomonas* classified sequences is similar in size to the expected size of *Pseudomonas aeruginosa*, so we expect that all or most of the contigs were correctly identified. In any case, assessment of genome quality and completeness is not part of SprayNPray’s pipeline and it is up to the user to assess their genome assemblies and metagenome bins.

We also used MetaBAT to generate bins from the contaminated *Pseudomonas* assemblies. MetaBAT generally performed well in creating bins containing *Pseudomonas* contigs. While the MetaBAT-generated *Pseudomonas* bins contained the majority of the contigs that are affiliated with *Pseudomonas* taxonomically (in terms of the number of contigs, as well as the total combined length of contigs), within these bins, we also identified contigs that are taxonomically affiliated with *Serratia*, the contaminant in these assemblies. Moreover, on all five sensitivity/specificity settings, one of the *Pseudomonas* assemblies was consistently split into two different bins.

### Case study: symbiont identification

SprayNPray can be used to extract bacterial symbiont contigs from an assembly that contains DNA from a variety of sources and domains of life. As an example, SprayNPray was run on an assembly of *Maconellicoccus hirsutus* (GCA_003261595; [25], the hibiscus mealybug, which contains two bacterial endosymbionts [26]. In this assembly, the majority of DNA is from the host insect. Visual inspection of the initial output of this assembly (Supplemental File 37) allowed for the identification of metrics (e.g. GC-content, top taxonomic hits, gene density) with which the software was re-run to generate two additional FASTA files, each corresponding to an individual endosymbiont (Supplemental Files 38 and 39).

### Case study: HGT identification

SprayNPray can be used to search eukaryotic contigs for genes that may have been horizontally/laterally obtained from bacteria via HGT. To showcase this functionality, we ran SprayNPray on an assembly of the citrus mealybug, *Planococcus citri* (de la Filia et al., [27], obtained from the MealyBugBase download server (https://download.mealybug.org/v1/Planococcus_citri_Pcitri.v1/fasta/dna/), and available for download here: 10.6084/m9.figshare.19184357 (Supplemental Files 40 and 41). This organism is known to encode multiple HGTs from bacteria on its nuclear genome [28]. A total of 519 putative HGTs were identified (Supplemental File 44), including those previously confirmed by Husnik et al., [28] (e.g. *murACDEF*, *bioABD*, *dapF*, *ddl*), as well as those that were reported but not confirmed (e.g. numerous AAA [ATPases Associated with diverse cellular Activities]-family ATPases of diverse origins, ankyrin repeat proteins with close homology to *Wolbachia* spp., and type III effectors) [28]. We note, however, that extreme caution should be taken in interpreting candidate HGTs,which should be validated by exploring the genomic context of each putative gene that is thought to have been horizontally acquired.

## Conclusions

Here, we present SprayNPray, a bioinformatics software designed to aid in the taxonomic analysis of diverse (meta)genomic datasets. The appeal of this software is its ease-of-use and straightforward output that is amenable to visual inspection and/or computational parsing. We designed this versatile software to lower barriers for those with limited experience in bioinformatics and programming.

### Availability and requirements

Project Name: SprayNPray.

Project home page: https://github.com/Arkadiy-Garber/SprayNPray

Operating system(s): Linux/MacOS.

Programming language: Python.

Other requirements: Python3, DIAMOND, Prodigal, MetaBAT.

License: GNU General Public License v3.0

Any restrictions to use by non-academics: No further restrictions to use beyond license.

## Supplementary Information

**Additional file 1: Supplemental Table 1.** GenBank assembly accessions for each genome included in the simulated genome, as well as the SprayNPray command line flags used to extract corresponding contigs.

**Additional file 2: Supplemental Figure 1.** Word clouds generated with SprayNPray, using R package "wordcloud," based on analysis of four of the MAGs from North Pond described in the case study on bin validation [19, 22]: **A**) NORP81, **B**) NORP91, **C**) NORP151, **D**) NORP148.

**Additional file 3: Supplemental File 1.** Modified summary output from a SprayNPray run on the simulated metagenome, showing the congruence of top taxonomic hits to each contig with their source genome.

**Additional file 4: Supplemental File 2.** FASTA file generated by SprayNPray, representing a subset of contigs from the simulated metagenome that match *Anaeromyxobacter*.

**Additional file 5: Supplemental File 3.** FASTA file generated by SprayNPray, representing a subset of contigs from the simulated metagenome that match *Archaeoglobus*.

**Additional file 6: Supplemental File 4.** FASTA file generated by SprayNPray, representing a subset of contigs from the simulated metagenome that match *Azorhizobium*.

**Additional file 7: Supplemental File 5.** FASTA file generated by SprayNPray, representing a subset of contigs from the simulated metagenome that match *Bradyrhizobium*.

**Additional file 8: Supplemental File 6.** FASTA file generated by SprayNPray, representing a subset of contigs from the simulated metagenome that match *Candidatus Kerfeldbacteria*.

**Additional file 9: Supplemental File 7.** FASTA file generated by SprayNPray, representing a subset of contigs from the simulated metagenome that match *Chlorobium*.

**Additional file 10: Supplemental File 8.** FASTA file generated by SprayNPray, representing a subset of contigs from the simulated metagenome that match *Ferrovum*.

**Additional file 11: Supplemental File 9.** FASTA file generated by SprayNPray, representing a subset of contigs from the simulated metagenome that match *Gallionellales*.

**Additional file 12: Supplemental File 10.** FASTA file generated by SprayNPray, representing a subset of contigs from the simulated metagenome that match Ignavibacteria.

**Additional file 13: Supplemental File 11.** FASTA file generated by SprayNPray, representing a subset of contigs from thesimulated metagenome that match *Nitrospira*.

**Additional file 14: Supplemental File 12.** FASTA file generated by SprayNPray, representing a subset of contigs from the simulated metagenome that match *Rickettsiales*.

**Additional file 15: Supplemental File 13.** FASTA file generated by SprayNPray, representing a subset of contigs from the simulated metagenome that match *Sulfuricella*.

**Additional file 16: Supplemental File 14.** FASTA file generated by SprayNPray, representing a subset of contigs from the simulated metagenome that match *Geobacter metallireducens*.

**Additional file 17: Supplemental File 15.** FASTA file generated by SprayNPray, representing a subset of contigs from the simulated metagenome that match *Geobacter sulfurreducens*.

**Additional file 18: Supplemental File 16.** FASTA file generated by SprayNPray, representing a subset of contigs from the simulated metagenome that match *Shewanella denitrificans*.

**Additional file 19: Supplemental File 17.** FASTA file generated by SprayNPray, representing a subset of contigs from the simulated metagenome that match *Shewanella oneidensis*.

**Additional file 20: Supplemental File 18.** FASTA file generated by SprayNPray, representing a subset of contigs from the simulated metagenome that match *Bacillus phage*.

**Additional file 21: Supplemental File 19.** FASTA file generated by SprayNPray, representing a subset of contigs from the simulated metagenome that match *Caulobacter phage*.

**Additional file 22: Supplemental File 20.** FASTA file generated by SprayNPray, representing a subset of contigs from the simulated metagenome that match *Sphaeroforma arctica*.

**Additional file 23: Supplemental File 21.** MetaBAT results after a run on the simulated metagenome.

**Additional file 24: Supplemental File 22.** SprayNPray output file after a run on a metagenome-assembled genome from North Pond (NORP81).

**Additional file 25: Supplemental File 23.** SprayNPray output file after a run on a metagenome-assembled genome from North Pond (NORP91).

**Additional file 26: Supplemental File 24.** SprayNPray output file after a run on a metagenome-assembled genome from North Pond (NORP151).

**Additional file 27: Supplemental File 25.** SprayNPray output file after a run on a metagenome-assembled genome from North Pond (NORP148).

**Additional file 28: Supplemental File 26.** SprayNPray output file after a run on a contaminated assembly of *Pseudomonas aeruginosa* isolate P32_36.

**Additional file 29: Supplemental File 27.** SprayNPray output file after a run on a contaminated assembly of *Pseudomonas aeruginosa* isolate P32_108.

**Additional file 30: Supplemental File 28.** SprayNPray output file after a run on a contaminated assembly of *Pseudomonas aeruginosa* isolate P41_119.

**Additional file 31: Supplemental File 29.** FASTA file generated by SprayNPray, representing a subset of contigs from the contaminated *Pseudomonas* P32_36 assembly that match *Pseudomonas aeruginosa*.

**Additional file 32: Supplemental File 30.** FASTA file generated by SprayNPray, representing a subset of contigs from the contaminated *Pseudomonas* P32_108 assembly that match *Pseudomonas aeruginosa*.

**Additional file 33: Supplemental File 31.** FASTA file generated by SprayNPray, representing a subset of contigs from the contaminated *Pseudomonas* P41_118 assembly that match *Pseudomonas aeruginosa*.

**Additional file 34: Supplemental File 32.** FASTA file generated by SprayNPray, representing a subset of contigs from the contaminated *Pseudomonas* P32_36 assembly that do not match *Pseudomonas aeruginosa*.

**Additional file 35: Supplemental File 33.** FASTA file generated by SprayNPray, representing a subset of contigs from the contaminated *Pseudomonas* P32_108 assembly that do not match *Pseudomonas aeruginosa*.

**Additional file 36: Supplemental File 34.** FASTA file generated by SprayNPray, representing a subset of contigs from the contaminated *Pseudomonas* P41_119 assembly that do not match *Pseudomonas aeruginosa*.

**Additional file 37: Supplemental File 35.** CheckM completeness and contamination scores for the FASTA files generated with SprayNPray after a run on the contaminated *Pseudomonas assemblies*.

**Additional file 38: Supplemental File 36.** MetaBAT results after a run on contaminated *Pseudomonas assemblies*.

**Additional file 39: Supplemental File 37.** SprayNPray output file after a run on an assembly of the mealybug *Maconellicoccus hirsutus*.

**Additional file 40: Supplemental File 38.** FASTA file generated by SprayNPray, representing a subset of contigs from an assembly of the mealybug *Maconellicoccus hirsutus* that match the symbiont Ca. *Tremblaya princeps*.

**Additional file 41: Supplemental File 39.** FASTA file generated by SprayNPray, representing a subset of contigs from an assembly of the mealybug *Maconellicoccus hirsutus* that match the symbiont Ca. *Doolittlea endobia*.

**Additional file 42: Supplemental File 40.** SprayNPray output file after a run on an assembly of the mealybug *Planococcus citri*.

**Additional file 43: Supplemental File 41.** SprayNPray output file after a run on an assembly of the mealybug *Planococcus citri*, with the -lvl flag set to Domain.

**Additional file 44: Supplemental File 42.** Candidate HGTs identified in the assembly of the mealybug *Planococcus citri*. Output file created with SprayNPray when the --hgt flag was included in the command.

## Data Availability

Supplemental files, corresponding to the genome data used and SprayNPray output, are provided with this article and at https://doi.org/10.6084/m9.figshare.19127084. These data are also freely available in the GitHub repository of this software: https://github.com/Arkadiy-Garber/SprayNPray/tree/master/Supplement-Case_Studies.

## Abbreviations

GC content: Guanine cytosine content, represented as a percentage of total base pairs
MAGs: Metagenome-assembled genomes
ORF: Open reading frame
NCBI: National Center for Biotechnology Information
nr: NCBI’s non-redundant protein database
BAM: Binary alignment map (file type)
AAI: Average amino acid identity
kb/kbp: Kilobase-pair
BLAST: Basic local alignment search tool
HGT: Horizontal gene transfer
