# Supplementary material for: SprayNPray: user-friendly taxonomic profiling of genome and metagenome contigs
Source: BMC Genomics. 2022 Mar 12;23:202. doi: 10.1186/s12864-022-08382-2 (PMC8917688; doi:10.1186/s12864-022-08382-2)
Supplement: Supplementary file 1 — Additional file 1: Supplemental Table 1. GenBank assembly accessions for each genome included in the simulated genome, as well as the SprayNPray command line flags used to extract corresponding contigs. [file 12864_2022_8382_MOESM1_ESM.docx]

| **Genome** | **Assembly Accession** | **Criteria (SprayNPray flag used to extract contigs)** |
| --- | --- | --- |
| *Archaeoglobus fulgidus* | GCF_000008665.1 | -genus Archaeoglobus |
| *Gallionellales bacterium* | GCA_001801025.1 | -genus Gallionellales |
| *Anaeromyxobacter dehalogenans* | GCF_000013385.1 | -genus Anaeromyxobacter |
| *Azorhizobium caulinodans* | GCF_000010525.1 | -genus Azorhizobium |
| *Bradyrhizobium japonicum* | GCF_000011365.1 | -genus Bradyrhizobium |
| *Chlorobium tepidum* | GCF_000006985.1 | -genus Chlorobium,Chlorobaculum |
| *Candidatus* Kerfeldbacteria | GCA_001818285.1 | -genus Kerfeldbacteria,Parcubacteria |
| *Ferrovum myxofaciens* | GCF_000735045.1 | -genus Ferrovum |
| *Nitrospira bacterium* | GCA_001303305.1 | -genus Nitrospira |
| *Ignavibacteria bacterium* | GCA_001802185.1 | -genus Ignavibacteria |
| *Geobacter sulfurreducens* | GCA_000007985.2 | -genus Geobacter -species sulfurreducens |
| *Geobacter metallireducens* | GCF_000012925.1 | -genus Geobacter -species metallireducens |
| *Shewanella denitrificans* | GCA_000013765.1 | -genus Shewanella -species denitrificans |
| *Shewanella oneidensis* | GCF_000146165.2 | -genus Shewanella -species oneidensis |
| *Rickettsiales bacterium* | GCA_000746585.2 | -genus Rickettsiales |
| *Sulfuricella denitrificans* | GCF_000297055.2 | -genus Sulfuricella |
| *Bacillus* phage | GCA_002758475.1 | --phage -genus Bacillus |
| *Caulobacter* phage | GCA_000899635.1 | --phage -genus Caulobacter |
| *Sphaeroforma arctica* | GCF_001186125.1 | --Eukaryota –CD 0.5 –GC 50 |
