# Supplementary material for: SprayNPray: user-friendly taxonomic profiling of genome and metagenome contigs
Source: BMC Genomics. 2022 Mar 12;23:202. doi: 10.1186/s12864-022-08382-2 (PMC8917688; doi:10.1186/s12864-022-08382-2)
Supplement: Supplementary file 2 — Additional file 2: Supplemental Figure 1. Word clouds generated with SprayNPray, using R package "wordcloud," based on analysis of four of the MAGs from North Pond described in the case study on bin validation [19, 22]: A) NORP81, B) NORP91, C) NORP151, D) NORP148. [file 12864_2022_8382_MOESM2_ESM.pdf]

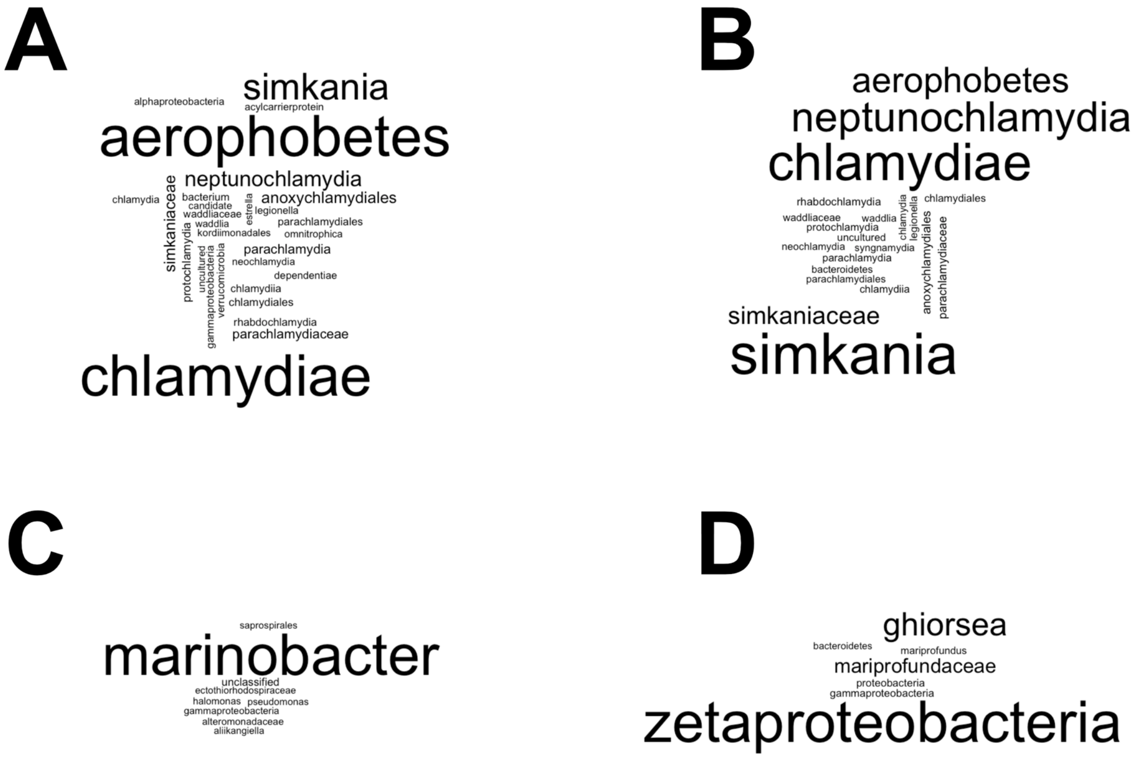

Supplemental Figure 1 | Word clouds generated with SprayNPray based on analysis of four of the MAGs from North Pond described in the case study on bin validation (Tully *et al*., 2018): A) NORP81, B) NORP91, C) NORP151, D) NORP148. This graphic is generated using the R package "wordcloud" (https://cran.r-project.org/web/packages/wordcloud).
